# Evaluation of Consistency of Treatment Response Across Regions—the LEADER Trial in Relation to the ICH E17 Guideline

**DOI:** 10.3389/fmed.2021.662775

**Published:** 2021-05-31

**Authors:** Henrik K. Nielsen, Stephanie DeChiaro, Bryan Goldman

**Affiliations:** ^1^Novo Nordisk A/S, Søborg, Denmark; ^2^Novo Nordisk Inc., Plainsboro, NJ, United States

**Keywords:** liraglutide, diabetes, regional, major adverse cardiovascular event, LEADER, ICH, E17, consistency

## Abstract

The US Food and Drug Administration in 2008 required new type 2 diabetes (T2D) medications to be subject to cardiovascular outcomes safety requirements. Accordingly, the global LEADER trial investigated cardiovascular outcomes of T2D treatment with liraglutide, a glucagon-like peptide-1 receptor agonist. LEADER (NCT01179048) was a multiregional clinical trial (MRCT) conducted from 2010 to 2016, thus completed before publication of the International Council for Harmonization (ICH) E17 guideline on MRCTs in 2017. Novo Nordisk pre-specified analysis of regional cardiovascular outcomes of LEADER participants. This paper assesses the pre-specified regional outcomes based on the ICH E17 guidelines on consistency evaluation. Regional LEADER participant numbers were broadly aligned with ICH E17 guidance and equally balanced across Europe, Asia, North America, and rest of the world. Overall primary major adverse cardiovascular events (MACE) composite outcome for the trial: hazard ratio (HR) (95% CI) 0.87 (0.78; 0.97); regional results varied, ranging from HR (95% CI) 0.62 (0.37; 1.04) (Asia) to 1.01 (0.84; 1.22) (North America). However, pre-specified Cox proportional-hazard regression analyses did not show clear evidence of interaction between regions and primary outcome (*p* = 0.20). Furthermore, *post hoc* analysis of the US population in the North American region found that adjusting for extrinsic or intrinsic factors did not account for this difference [HR (95% CI) 1.03 (0.84; 1.25)]. LEADER data evaluation demonstrated general consistency in cardiovascular safety across regions, except for US participants. Discrepancies in the North American region may relate to drug exposure or chance, but, as these were *post hoc* findings, the overall primary result is valid, aligned with ICH E17 guidelines.

## Introduction

Previous literature has indicated a potential association between certain diabetes medications and increased cardiovascular risk (1, 2). This prompted action from the US Food and Drug Administration (FDA), which, in 2008, issued guidance for sponsors of new type 2 diabetes (T2D) medication to demonstrate a cardiovascular risk ratio below 1.8 pre-approval and ultimately below 1.3 post-approval (3). Liraglutide, a glucagon-like peptide-1 (GLP-1) receptor agonist, received FDA approval in 2010 to improve glycemic control in adults with T2D, with a post-marketing requirement to conduct a randomized, double-blind, controlled trial evaluating the effect of liraglutide on the incidence of major adverse cardiovascular events (MACE). Novo Nordisk, which developed liraglutide, therefore undertook the global cardiovascular outcomes trial (CVOT) LEADER (Liraglutide Effect and Action in Diabetes: Evaluation of Cardiovascular Outcome Results), recruiting 9,340 participants from 32 countries. The trial (ClinicalTrials.gov NCT01179048) was initiated in 2010 and the results became available in 2016 (4).

In response to increasing globalization of drug development, the International Council for Harmonization (ICH) issued a final harmonized guideline in November 2017 titled “E17 General Principles for Planning and Design of Multi-Regional Clinical Trials,” which aimed to increase the acceptability of multiregional clinical trials (MRCTs) in global regulatory submissions (5). Among other topics, this document provides guidance on regional sample size allocation and examination of consistency of outcomes across regions and subpopulations.

The objective of this paper was to evaluate the consistency of cardiovascular outcomes following liraglutide treatment across regions studied in the LEADER clinical trial, in relation to the ICH E17 guideline principles for consistency evaluation.

## Materials and Methods

The LEADER clinical trial design and methods have been published previously (6). LEADER was a multicenter, double-blind, placebo-controlled clinical trial performed at 410 sites in 32 countries. Participants with T2D and a high risk of cardiovascular disease were randomized 1:1 to liraglutide or placebo, both in addition to standard of care (6). Participants were followed for at least 3.5 years. The primary endpoint was the time from randomization to a composite MACE outcome consisting of first occurrence of cardiovascular death, non-fatal myocardial infarction (MI), or non-fatal stroke. Secondary endpoints included the first occurrence of an expanded composite cardiovascular outcome, including cardiovascular death, non-fatal MI, non-fatal stroke, revascularization, hospitalization for unstable angina, or hospitalization for chronic heart failure. Participants were allowed to stop and restart their study medication while remaining in the trial; this is common practice in CVOTs to maximize participant retention. The trial protocol was approved by the institutional review board or ethics committee at each participating center and all the patients provided written informed consent. Further details on the ethics committees can be found in the primary manuscript (4).

A central external event adjudication committee performed independent and blinded adjudication of the primary endpoint events.

### Statistical Analysis

All time-to-event endpoints in LEADER were analyzed using a Cox proportional-hazard regression model. For the primary endpoint of time to first MACE, a hierarchical testing strategy was used for the liraglutide group vs. the placebo group, first testing for non-inferiority and subsequently for superiority. Non-inferiority was established for the primary outcome if the upper limit of the two-sided 95% confidence interval (CI) of the hazard ratio (HR) was <1.30, and superiority was established if the upper limit was <1.00 (4).

Pre-specified subgroup analyses were performed to investigate any potential differences between regional subpopulations with respect to the primary endpoint. A number of additional *post hoc* subgroup analyses were performed to elucidate these differences, exploring whether the results may be explained by differences in any intrinsic (demographic, baseline characteristics, and cardiovascular history at screening) or extrinsic (concomitant medication) factors (Table 1). Regional differences were further explored using a shrinkage estimation procedure (7), as well as the Gail-Simon test for qualitative interaction (8).

**Table 1:** Characteristics evaluated as potential contributors for impact on time to first MACE in US population and non-US populations.

**Demographics**	**Baseline characteristics**	**Concomitant medications at baseline**	**Cardiovascular history and complications at screening**
Age	BMI	Antidiabetic medication^a^	Cardiovascular risk
Gender	Body weight	Antihypertensive medication^b^	Prior MI
Smoking status	Systolic blood pressure	Diuretics	Prior PCI
Race	Diastolic blood pressure	Lipid-lowering drugs	Prior hypertension
T2D duration	Heart rate	Platelet aggregation inhibitors	Prior TIA
	HbA_1c_	Antithrombotic medication	Prior ischemic heart disease
	LDL cholesterol		Prior left ventricular diastolic dysfunction
	HDL cholesterol		Prior carotid artery stenosis
	Total cholesterol		Prior >50% stenosis
	Triglycerides		Peripheral arterial disease
	Renal function		CABG
	Albuminuria		

*Each parameter has been analyzed in a Cox model with treatment, the parameter and its interaction with treatment, and the factor US/non-US and its interaction with treatment.*

*^a^Includes the following categories: “1 OAD,” “more than 1 OAD(s),” “insulin + OAD(s),” “insulin – OAD(s),” and “none.”*

*^b^Includes the following categories: “beta-blockers,” “calcium channel blockers,” “loop diuretics,” “renin system blockers,” and “other.”*

*BMI, body mass index; CABG, coronary artery bypass graft; HbA_1c_, glycated hemoglobin; HDL, high-density lipoprotein; LDL, low-density lipoprotein; MACE, major adverse cardiovascular events; MI, myocardial infarction; OAD, oral antidiabetic drug; PCI, percutaneous coronary intervention; T2D, type 2 diabetes; TIA, transient ischemic attack; US, United States*.

## Results

The LEADER clinical trial was well conducted: 96.8% of participants completed their final visit and vital status was known for 99.7% of the participants. The primary composite outcome of 3-component MACE occurred in fewer participants (%) in the liraglutide group [608 of 4,668 participants (13.0%)] than in the placebo group [694 of 4,672 (14.9%)], with an HR (95% CI) of 0.87 (0.78; 0.97). The two-sided *p*-values for non-inferiority (risk ratio below 1.3) and for superiority (risk ratio below 1.0) were *p* < 0.001 and *p* = 0.01, respectively. There was strong consistency between the results for the primary endpoint and those obtained in various secondary endpoints (4).

**Figure 1:**
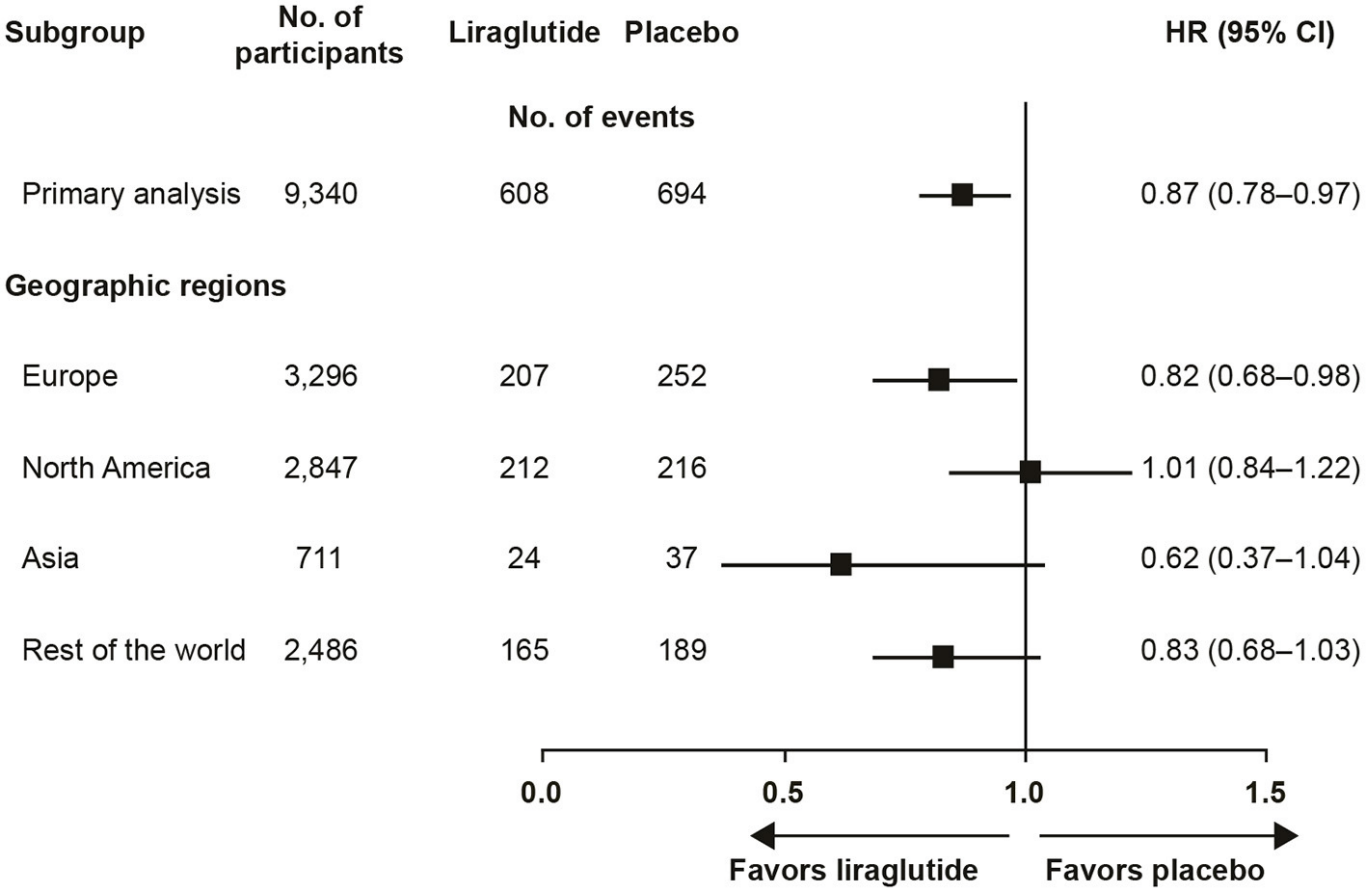
LEADER primary analysis by geographic region (4). CI, confidence interval; HR, hazard ratio.

The trial recruited participants globally and the pre-defined regions were (number of participants in parenthesis): Europe (3,296), North America (2,847), Asia (711), and rest of the world (2,486). The outcome varied by region from a HR (95% CI) of 0.62 (0.37; 1.04) in Asia to 1.01 (0.84; 1.22) in North America (Figure 1) (4). Pre-specified Cox proportional-hazard regression analyses, performed for regional participant populations with respect to the primary outcome, did not show clear evidence of interaction between the geographic region and the primary outcome (*p* = 0.20). Further *post hoc* evaluation of the results in North America found HR (95% CI) estimates of 1.03 (0.84; 1.25) for the US and 0.80 (0.42; 1.52) for Canada. This observation prompted further investigations of the US population, the largest country in the region, comprising 88% of the North American population in the study (6).

**Figure 2:**
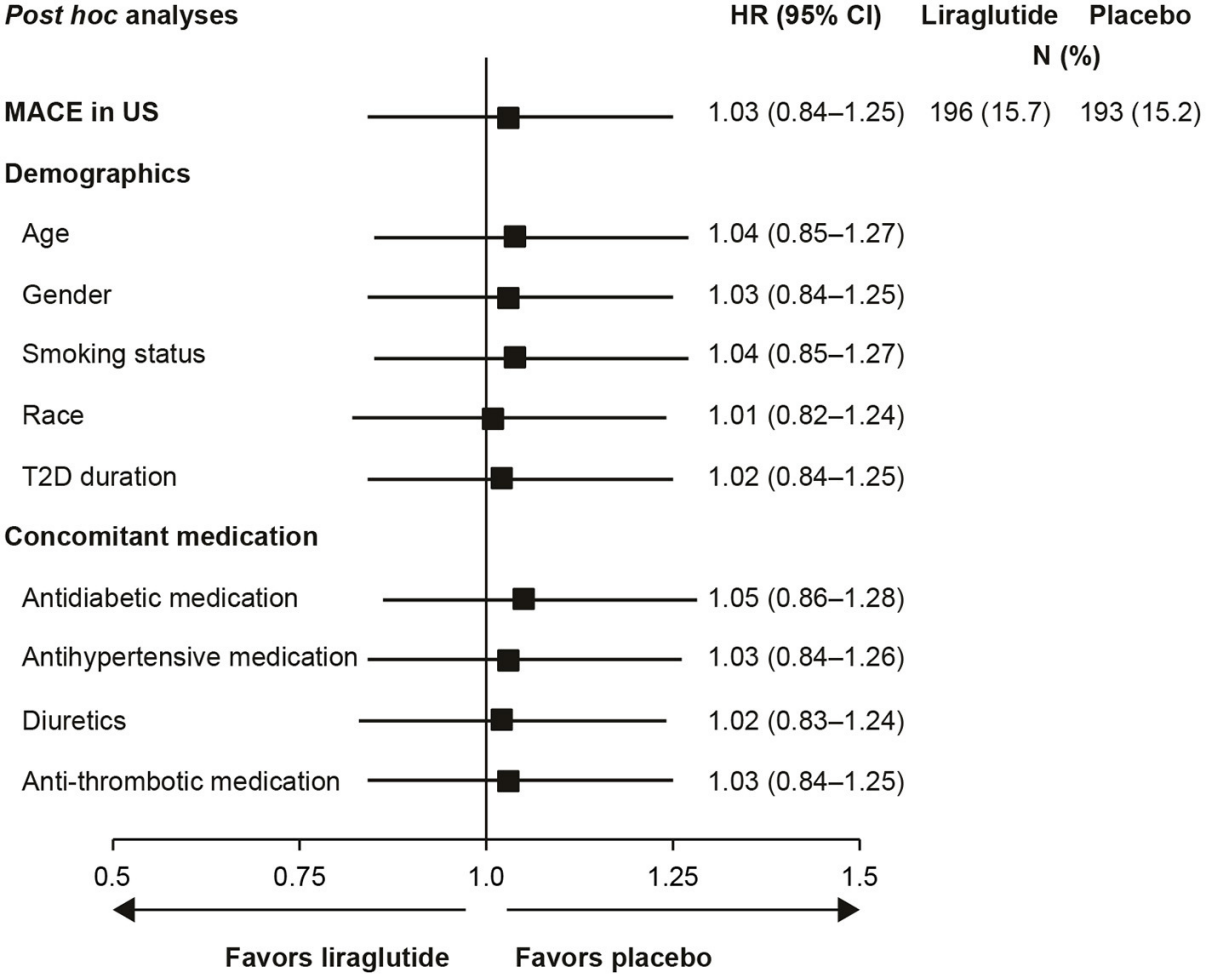
Analysis of time to first MACE in US participants with adjustment for baseline demographics and concomitant medications (29). Full analysis set; HR with 95% CI. Each row presents results of a Cox proportional hazards model with treatment as factor and adjustment for each baseline demographic variable or concomitant medication. All HRs are for the comparison of liraglutide to placebo. CI, confidence interval; HR, hazard ratio; MACE, major adverse cardiovascular event; N, number of patients with an event; T2D, type 2 diabetes; US, United States.

Additional *post hoc* analyses found that adjusting for intrinsic or extrinsic factors had little effect on the US outcomes (Figure 2). In addition, blood glucose control, as measured by HbA_1c_ over time, did not account for the US outcomes (data not shown).

**Figure 3:**
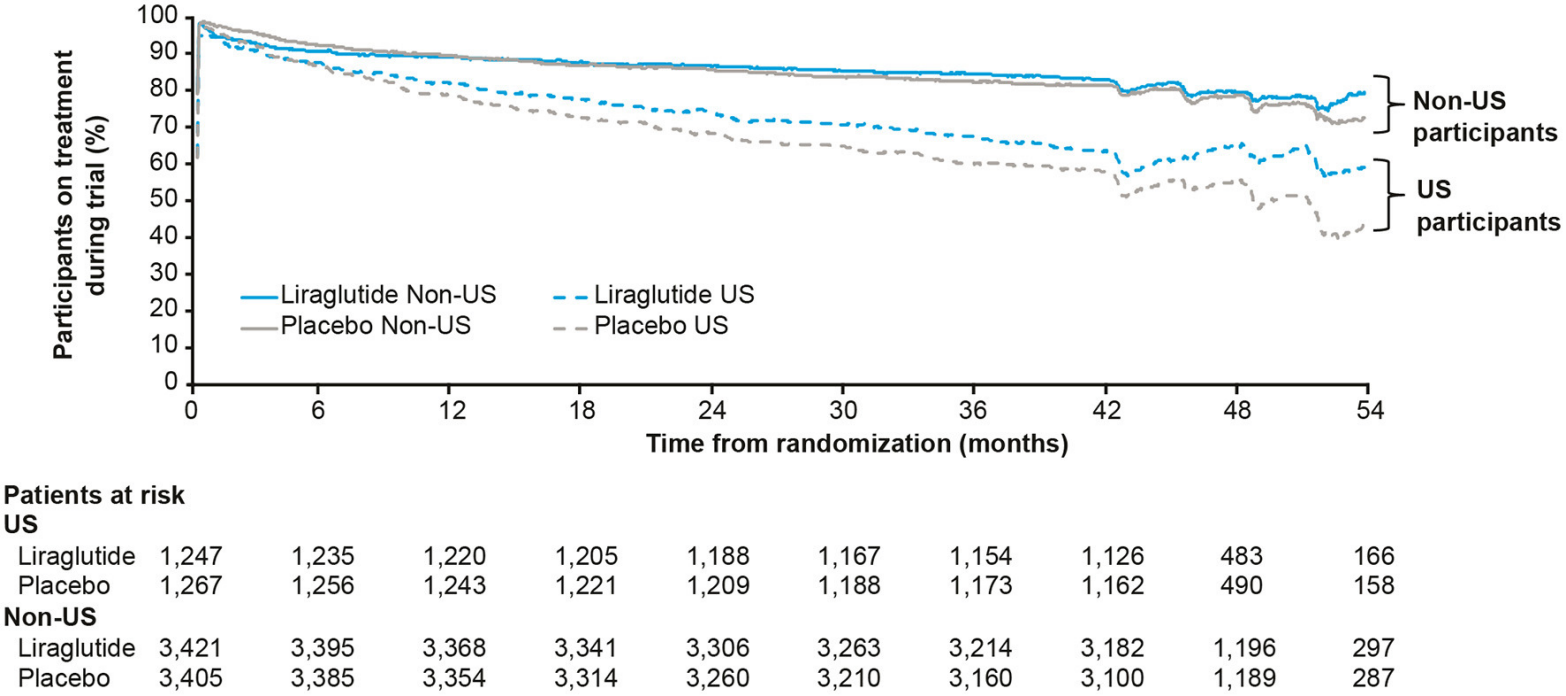
Percentage of US and non-US participants on treatment with liraglutide or placebo during the LEADER trial (30). Full analysis set. US, United States.

Participants could stop and restart their study medication throughout the trial; it was found that the US participants were less adherent than non-US participants to study drug (Figure 3). *Post hoc* analysis of MACE while the US participants were on-treatment gave an HR (95% CI) of 0.89 (0.69; 1.14), close to the global on-treatment result of 0.83 (0.73; 0.95). However, since this analysis involves adjustment for events occurring after randomization, it remains unclear whether the neutral cardiovascular result in the US can be explained by lower exposure to study medication.

## Discussion

The effect of liraglutide is thought to modify the progression of atherosclerotic vascular disease, without variation between racial or regional populations (4, 9). In our study, the North American population accounted for 30% of the total study population, which is broadly in line with the ICH E17 recommendation (5). The LEADER trial was conducted as a regulatory requirement for the FDA and was also in alignment with European Medicines Agency (EMA) regulatory requirements.

Other trials have also shown differences in results according to country or region. Yusuf and Wittes analyzed geographic variations in the results of nine randomized clinical trials (10). Possible explanations discussed by Yusuf and Wittes included differences in standard of care, concomitant medication, geographical differences in the disease parasite, underlying risk factors, enrolment differences, or chance (10). In the PLATO trial (11), investigating ticagrelor vs. clopidogrel for patients with acute coronary syndrome, the North American population had an HR (95% CI) of 1.25 (0.93; 1.67) compared to the overall observed benefit [HR = 0.84 (0.77; 0.92)]; the *p*-value for interactions was 0.05. Further investigation showed that higher aspirin doses seemed to reduce or even inverse the positive effect of ticagrelor (12) and, as the US participants were taking higher doses of aspirin, this was believed to be the explanation. Ticagrelor received a boxed warning for its US label against concomitant used of aspirin above 100 mg, although this was not supported by the FDA advisory committee and has been disputed by several authors (10, 12).

CVOTs with other GLP-1 receptor agonists have also reported higher HRs in the population from North America as compared to the overall population. A meta-analysis of controlled trials investigating cardiovascular endpoints of patients treated with a GLP-1 receptor agonist provided an HR (95% CI) of 0.94 (0.85; 1.04) in North America compared to the overall result of 0.85 (0.78; 0.93) (13). A marginally significant interaction (*p* = 0.05) was detected for this region. The highest observed HR in North America (1.14) was observed in the REWIND trial with dulaglutide (14); however, in this case, the 95% CI also included unity (0.89; 1.47). In the CVOT for empagliflozin, a sodium-glucose cotransporter-2 (SGLT-2) inhibitor to treat diabetes, a neutral HR of 1.02 (0.81; 1.28) was observed in Europe, the largest participating region, whereas the HR for the total trial population was 0.86 (0.74; 0.99) (15).

Ferreira et al. investigated geographical variation in a heart failure trial (16) in which North American participants responded well to treatment whereas Eastern Europe participants showed HRs of ≥1.0. Marked differences in baseline conditions and difficulties in standardizing the acute treatment may have contributed to the observations. Kristensen et al. conducted many *post hoc* subgroup analyses (17) of the PARDIGM-HF (heart failure) trial and, although many differences were reported, the overall outcome of this trial was consistent across regions.

### Statistical Considerations

The challenges of testing for quantitative interaction (i.e., differences in the magnitude of treatment effect among subgroups) are well-described (18). Such testing is known to suffer from low power, especially when many subgroup differences are tested and adjustment for multiple comparisons is needed. Furthermore, as noted by Gail and Simon (8), the subgroup differences of greatest clinical importance are those in which the direction of the treatment effect is different for different subgroups. Power for such qualitative interaction tests is even lower. In LEADER, the *post hoc* test for quantitative interaction of the US vs. non-US subgroups was nominally significant (*p* = 0.049), although this result was not corrected for multiple comparisons. The Gail-Simon test found no evidence of qualitative interaction between these subgroups (*p* = 0.40).

Shrinkage estimation has been proposed as an analytic tool to further explore regional differences in MRCTs (3, 7, 19). This method estimates the regional treatment effect as a weighted average of the overall treatment effect and that observed directly based on the data for each region. The differences among regional treatment effects are thereby shrunk in proportion to the uncertainty in the estimates from the within-region analyses. During their evaluation of the LEADER result, the FDA applied a Bayesian shrinkage estimation procedure to the analysis of time to first MACE by region (20), the results of which are shown in Table 2. As expected, the results of this analysis show regional estimates of treatment effect closer to the overall mean than the pre-specified subgroup analyses, with the amount of shrinkage of regional treatment effect estimates toward the overall estimate positively associated with the amount of uncertainty within each region. For example, the 95% CI for the subgroup analysis of participants from Asia is widest; this is also the region in which most shrinkage is observed. These results suggest that regional differences in treatment effect are much smaller than suggested by the analyses of populations by region, and do not appear to be clinically meaningful. It thereby supports the conclusion that the overall estimate of treatment benefit applies across all regions included in the LEADER trial.

**Table 2:** Bayesian shrinkage estimation of time to first MACE by region (20).

	**Sample estimate**		**Bayes shrinkage estimate**	
**Region**	**HR**	**95% CI**	**HR**	**95% CI**
Asia	0.62	0.37, 1.04	0.80	0.59, 1.09
Europe	0.82	0.68, 0.98	0.84	0.71, 0.98
North America	1.01	0.83, 1.22	0.94	0.79, 1.12
Rest of the world	0.83	0.68, 1.03	0.85	0.72, 1.00

*Regional sample estimates obtained from a Cox regression model with treatment, region, and its interaction with treatment.*

*CI, confidence interval; HR, hazard ratio; MACE, major adverse cardiovascular events*.

### Regulatory Considerations

The FDA convened an advisory committee meeting to discuss the LEADER results. In its briefing presentation on the subgroup analyses, the FDA reported the following (21):

- In summary, point estimates of the HRs were above 1.0 for the US subgroups and for participants older than 60 years with risk factors.
- This could suggest possible inconsistency in the effect for MACE across these subgroups.
- Several analyses were conducted to explain these findings, but it is important to emphasize that these were exploratory and there still remains a possibility that the subgroup findings could be explained by chance alone.

The advisory committee voted 17–2 to support the notion that LEADER provides substantial evidence that liraglutide reduces cardiovascular risk in patients with T2D. The committee members voiced their confidence in this decision based on the primary MACE results, as well as the consistent trend in the individual components of MACE. Members noted that, although the subgroup findings described above were notable, they did not refute the overall LEADER results. Subsequently, the FDA approved the additional indication “to reduce the risk of major adverse cardiovascular events in adults with type 2 diabetes mellitus and established cardiovascular disease.” The data section shows the Kaplan–Meier plot for the primary endpoint; no subgroup analyses are included in the label (22).

Many health authorities around the world have now approved inclusion of the LEADER data in the label and, in most cases, an additional indication has been granted (22–26). Only the Chinese health authorities requested inclusion of local subgroup analyses (Table 3). The MACE and expanded MACE results for a total of 14 MACEs in 92 Chinese participants in the trial were included. In Japan, liraglutide is approved in lower doses than the rest of the world, primarily due to how the original development program was designed (28). Furthermore, no Japanese participants were included in the LEADER trial. Based on this, the Pharmaceuticals and Medical Devices Agency (PMDA) did not want to include the LEADER data in the Japanese label. ICH E17 guidance does allow inclusion of multiple doses in an MRCT; if designed today, this may have been a consideration for the LEADER trial.

**Table 3:** Regulatory approvals of LEADER in the Victoza® label.

**Health authority**	**Indication**	**Kaplan–Meier for MACE**	**CV events Forest plot**	**Regional subgroup analyses**	**Approval year**
FDA (US) (22)	√	√	X	X	2017
EMA (EU) (24)	Expanded diabetes	√	√	X	2017
Health Canada (Canada) (23)	√ (CV death)	√	√	X	2017
TGA (Australia) (26)	√	√	√	X	2018
SwissMedic (Switzerland) (25)	√	√	MACE only	X	2018
NMPA (China)	√	√	√	√	2018/2020
CDE (Taiwan) (27)	√	√	X	X	2018

*CDE, Center for Drug Evaluation; CV, cardiovascular; EMA, European Medicines Agency; EU, European Union; FDA, US Food and Drug Administration; MACE, major adverse cardiovascular events; NMPA, National Medical Products Administration; TGA, Therapeutic Goods Administration; US, United States. √, included; X, not included*.

The LEADER trial had a high-quality study design with elements aligned to the ICH E17 guideline on general principles for planning and design of MRCTs. Due to FDA and EMA regulatory commitments, as well as the feasibility of including sites with the capabilities and experience necessary to conduct outcomes trials, about two-thirds of participants were recruited in Europe and North America. Today, a more even global distribution would be preferred when conducting a CVOT in diabetes.

## Conclusion

The LEADER trial was a MRCT designed along the lines of ICH E17 and its conduct provided robust data for assessment of cardiovascular safety and benefit for liraglutide. There was general consistency of findings across sensitivity subgroup and subpopulation analyses that further support the primary analysis. The discrepancy of findings in the North American region and US subpopulations may be due to lower US drug exposure or chance.

When pre-planned regional and subpopulation analyses reveal surprising regional differences, supplemental *post hoc* analyses should be performed. Unless plausible and meaningful differences are revealed, the global primary result is valid for all regions. This is consistent with the ICH E17 basic principles and was implemented by regulators around the world.

## Data Availability Statement

The original contributions presented in the study are included in the article/supplementary material; further inquiries can be directed to the corresponding author.

## Ethics Statement

The trial protocol was approved by the institutional review board or ethics committee at each participating center and all the patients provided written informed consent. Further details on the ethics committees can be found in the primary manuscript (4).

## Author Contributions

HKN contributed with the global input and supervised the global regulatory processes/work. SD helped with the US perspective, having worked on the US submission/approval of the LEADER supplement. BG contributed to the conception/design of the work and the interpretation of data, and providing statistical input. All authors contributed to drafting, revising and review of the publication, and take responsibility for the integrity of the work as a whole and have given final approval for the version to be published.

## Conflict of Interest

HKN and BG are employees and shareholders of Novo Nordisk A/S. SD is an employee of Novo Nordisk, Inc. and a shareholder of Novo Nordisk A/S. Sponsorship for this analysis and article processing charges were funded by Novo Nordisk A/S, which also had a role in the design, analysis and reporting of the trial.
